# Myocardial characterization in pre-dialysis chronic kidney disease: a study of prevalence, patterns and outcomes

**DOI:** 10.1186/s12872-019-1256-3

**Published:** 2019-12-16

**Authors:** Anna M. Price, Manvir K. Hayer, Ravi Vijapurapu, Saad A. Fyyaz, William E. Moody, Charles J. Ferro, Jonathan N. Townend, Richard P. Steeds, Nicola C. Edwards

**Affiliations:** 1 Birmingham Cardio-Renal Group, Institute of Cardiovascular Sciences, University of Birmingham, Birmingham, UK; 2 Department of Nephrology, Queen Elizabeth Hospital , Birmingham, UK; 3 Department of Cardiology, Queen Elizabeth Hospital, Birmingham, UK; 4 Green Lane Cardiovascular Service, Auckland City Hospital, Birmingham, UK

**Keywords:** Cardiac magnetic resonance, Gadolinium, Chronic kidney disease, Fibrosis

## Abstract

**Background:**

Late gadolinium enhancement (LGE) using cardiac magnetic resonance (CMR) characterizes myocardial disease and predicts an adverse cardiovascular (CV) prognosis. Myocardial abnormalities, are present in early chronic kidney disease (CKD). To date there are no data defining prevalence, pattern and clinical implications of LGE-CMR in CKD.

**Methods:**

Patients with pre-dialysis CKD (stage 2–5) attending specialist renal clinics at University Hospital Birmingham (UK) who underwent gadolinium enhanced CMR (1.5 T) between 2005 and 2017 were included. The patterns and presence (LGEpos) / absence (LGEneg) of LGE were assessed by two blinded observers. Association between LGE and CV outcomes were assessed.

**Results:**

In total, 159 patients received gadolinium (male 61%, mean age 55 years, mean left ventricular ejection fraction 69%, left ventricular hypertrophy 5%) with a median follow up period of 3.8 years [1.04–11.59]. LGEpos was present in 55 (34%) subjects; the patterns were: right ventricular insertion point *n* = 28 (51%), mid wall *n* = 18 (33%), sub-endocardial *n* = 5 (9%) and sub-epicardial *n* = 4 (7%). There were no differences in left ventricular structural or functional parameters with LGEpos. There were 12 adverse CV outcomes over follow up; 7 of 55 with LGEpos and 5 of 104 LGEneg. LGEpos was not predicted by age, gender, glomerular filtration rate or electrocardiographic abnormalities.

**Conclusions:**

In a selected cohort of subjects with moderate CKD but low CV risk, LGE was present in approximately a third of patients. LGE was not associated with adverse CV outcomes. Further studies in high risk CKD cohorts are required to assess the role of LGE with multiplicative risk factors.

## Background

Patterns of late gadolinium enhancement (LGE) identified by cardiac magnetic resonance (CMR) imaging are integral to characterizing myocardial disease and are predictive of adverse cardiovascular (CV) outcome in both ischaemic and non-ischaemic disease [1, 2] LGE correlates with expansion of the extracellular matrix of the myocardium through perivascular and replacement fibrosis and predicts increased mortality and CV hospitalisation independently of other routinely assessed CMR parameters including left ventricular ejection fraction (LVEF) [1–3]. Furthermore, the absence of mid-wall fibrosis in non-ischaemic cardiomyopathy has been associated with improved reverse LV (left ventricular) remodelling following cardiac resynchronisation therapy and better long-term survival with both severe and mild-moderate degrees of systolic impairment [1, 2, 4]. Left ventricular hypertrophy (LVH), dilatation and systolic impairment constitute “uremic cardiomyopathy” and are thought to explain the disproportionate rates of heart failure and sudden death which constitute almost 50% of deaths in end-stage kidney disease (ESKD) compared to only 6.7% from myocardial infarction / ischaemia [5, 6]. Myocardial interstitial fibrosis is present on endomyocardial biopsy in ESKD and on CMR late gadolinium enhancement imaging (LGE-CMR) with sub-endocardial (14%) and mid-wall patterns (14%) in patients without diagnosed CV disease [7, 8]. Subsequent observational CMR studies have confirmed that myocardial abnormalities of systolic deformation and interstitial fibrosis are detectable from the earliest stages of CKD, before development of LVH, or a reduction in LVEF, emphasising the potential importance of myocardial disease in driving adverse outcomes in this cohort [9]. The significance of LGE in CKD remains unknown.

## Methods

### Study design and population

A retrospective study of patients with pre-dialysis CKD (stages 2–5; estimated glomerular filtration rate (eGFR) 90 to < 15 ml/min/1.73m^2^) undergoing CMR as part of clinical research studies between 2005 and 2017, see Additional file 1: Table S1 for details [9, 10]. Patients provided consent to take part and allow retrospective follow up. Detailed inclusion / exclusion study criteria and patient clinical characteristics are outlined in Additional file 1: Table S1. Patients were recruited from studies investigating the changes in non-ischaemic myocardial structure and function, including fibrosis, in early CKD. All patients were recruited from specialist renal clinics at a tertiary unit in Birmingham, UK and were followed up regularly. Trial exclusion criteria included; diagnosed coronary/ peripheral artery disease, LVEF < 50%, and diabetes, with the aim of reducing possible confounding effects of atheromatous disease.

### Data collection

Patient demographics, haematological and biochemical data were obtained from electronic patient records system (Clinical Portal©). Renal aetiology was confirmed on imaging or biopsy studies and eGFR calculated by the creatinine based Modification of diet in renal disease (MDRD) equation at baseline [11].

### Cardiac magnetic resonance protocol

CMR (1.5 T, MAGNETOM Symphony and Avanto: Siemens, Erlangen, Germany) was performed to assess LV and right ventricular (RV) volumes, function, and LV mass using standard breath-hold steady state free precession sequences. Gadolinium contrast (Magnevist®, Gadovist®) at a dose 0.15–0.2 mmol/Kg was administered to subjects with eGFR > 15 ml/min/1.73m^2^ between 2005 and 2012 and > 30 ml/min/1.73m^2^ between 2012 and 2017 following a change in Medicines and Healthcare Products Regulatory Agency guidelines. Standard T1-weighted gradient echo inversion recovery images were performed at 7–10 min after contrast in standard long axis and short axis stack imaging.

### CMR analysis

**Fig. 1:**
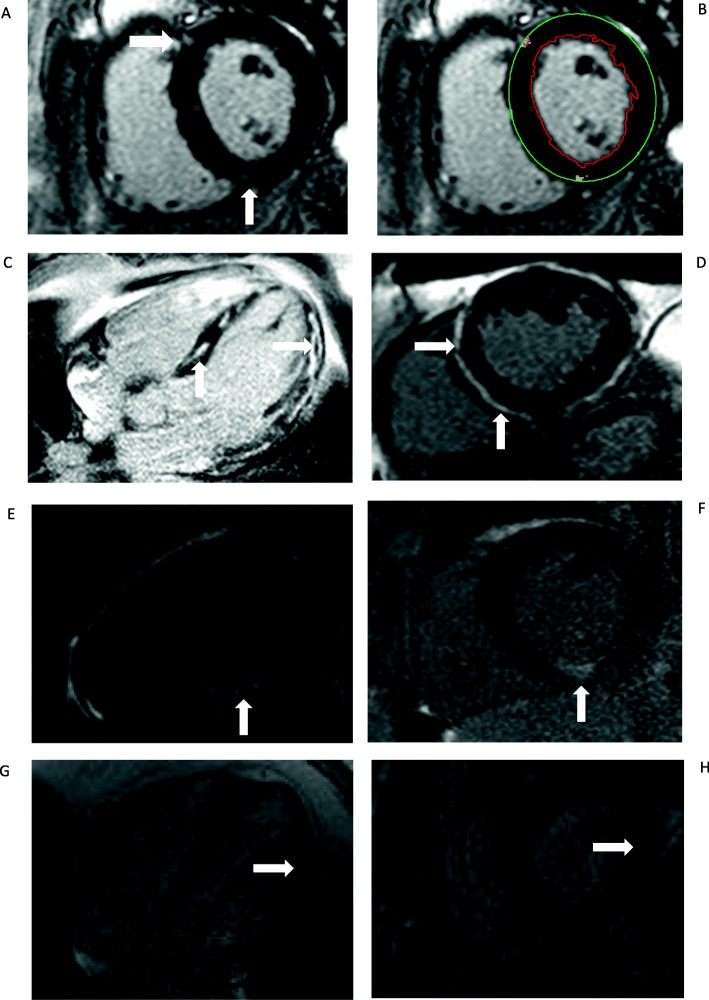
Examples of LGEpos patterns and quantification methods. **a** & **b** A mid-ventricular LGE image with inferior and superior RVIP LGE (**a**). Endocardial and epicardial contours are drawn using cvi42 (**b**) and the maximum area of hyper enhancement selected. Areas of LGE are quantified using full-width-half maximum methodology and appear pixelated. LGE is given as grams and represented as percentage of total left ventricular mass. This patient had membranous nephropathy, stage 2 CKD and was in remission at the time of CMR. **c** & **d** Mid-wall LGE; Horizontal long axis 4-chamber view (**c**) and corresponding mid short axis view (**d**) showing extensive septal and lateral mid wall LGE in a patient with CKD secondary to Adult Polycystic Kidney Disease. The patient initially underwent a CMR as part of a research study and was asymptomatic. Coronary angiogram was normal. Ambulatory ECG monitoring demonstrated sinus rhythm with no arrhythmias. He later had a successful renal transplant with no arrhythmias or deterioration in LV function. Follow up CMRs have confirmed fixed appearances of LGE. **e** & **f** Sub-endocardial; Vertical long axis 2-chamber (**e**) and corresponding short axis view (**f**) demonstrating focal inferior sub-endocardial infarction.* The patient had focal segmental glomerular sclerosis with stable CKD stage 3 disease. The patient was asymptomatic. He underwent a CT coronary angiogram and calcium score which demonstrated moderate coronary calcification above the 90th centile for age and gender but no evidence of coronary artery stenosis. **g** & **h** Sub-epicardial LGE; Horizontal long axis 4-chamber view (**g**) and corresponding basal short axis view (**h**) with sub-epicardial LGE in the basal-mid inferior wall. The patient was a 59 year old with Adult Polycystic Kidney Disease and stable CKD stage 3a. He was asymptomatic. There has been no clinical history suggestive of either sarcoidosis or myocarditis. * Images **e** & **f** were originally published in QJM: An International Journal of Medicine [15]

Offline analysis was performed by experienced operators (NE, AP) using cvi42® software (version 5.3.4 cvi42, Circle Vascular Imaging, Canada). Analysis of LV function, volume and LV mass was performed with delineation of papillary muscles and trabeculations using thresholding [12]. LV hypertrophy was defined based on age and gender [13]. The presence and pattern of LGE was assessed qualitatively and quantitatively by both observers and defined as LGEpos if present on two or more contiguous short axis slices or on corresponding long axis images with appropriate phase swapping. Patterns were defined as; sub-endocardial, diffuse / mid wall, sub-epicardial and focal right ventricular insertion point (RVIP) according to previous published descriptions [14]. Quantification of LGE was determined using full width half max methodology and expressed as the total percentage of the LV mass, see Fig. 1 [16].

### Follow up

Follow-up was defined from the date of CMR to the date of either the last outpatient clinic episode or the last known inpatient admission. Right censoring was used for survival analysis. Adverse events were recorded from electronic patient records systems. Major adverse CV clinical outcomes were defined as death from CV disease, myocardial infarction, stroke, peripheral vascular disease, or hospital admission with heart failure. Cause of death was classified according to 1a and 1b on the death certificate. Total admissions, renal admissions (defined as routine management of CKD such as intravenous iron infusions, access surgery or admissions related to transplantation) and non-renal admissions were recorded.

### Statistical analysis

Statistical analysis was performed using IBM SPSS© version 23 Armonk, New York. Significance was classified as a *p* value < 0.05. Continuous parametric variables were analysed using unpaired t tests (age, eGFR, blood pressure, body mass index, ventricular volumes and mass), non-parametric data such as N-terminal pro b-type natriuretic peptide (NTproBNP) was analysed after the data was logged. A one-way analysis of variance was used for continuous parametric variables with more than two groups. Binary variables such as immunosuppressive therapy, anti-hypertensive therapy, sex, presence of left ventricular hypertrophy and statin use were analysed using Fisher’s exact tests. Chi square tests were used if there were several categories such as aetiology of renal disease. Kaplan Meier survival curves with Breslow and Log rank analysis were used to compare survival between those with and without LGE. Binary logistic regression was used to determine the influence of each variable on the presence of LGE. The results of the univariate analysis were displayed graphically as a forest plot. Forward regression was subsequently used for multivariate analysis. Absolute mean bias and interclass correlation coefficients were used to determine intra and inter observer variability.

## Results

In total 159 patients underwent LGE-CMR with LGEpos present in 55 /159 subjects (34%). None of the 43 healthy controls had evidence of LGE [9]. Patient characteristics are presented in Table 1. There were no differences in demographics or blood pressure. Median follow up was 3.8 years [1.04–11.59].

**Table 1:** Baseline patient characteristics according to presence or absence of late gadolinium enhancement on cardiac MRI

	**LGEpos*n*= 55**	**LGEneg*n*= 104**	***P*value**
Age (years)	57 ± 12	53 ± 13	0.069
Male sex *n* (%)	38 (69)	59 (57)	0.171
BMI	28 ± 4	27 ± 4	0.088
eGFR (ml/min/1.73m^2^)	54 ± 15	52 ± 16	0.502
KDIGO stage *n* (%)			0.649
2 (eGFR 60–89 ml/min/1.73m^2^)	22 (40)	32 (31)	
3a (eGFR 45–59 ml/min/1.73m^2^)	17 (30)	35 (33)	
3b (eGFR 30–44 ml/min/1.73m^2^)	14 (26)	29 (28)	
4 (eGFR 15–29 ml/min/1.73m^2^)	2 (4)	8 (8)	
Aetiology *n* (%)			0.117
Vasculitis	7 (13)	9 (9)	
GN	15 (27)	49 (47)	
Hereditary	10 (18)	13 (12)	
Systemic	8 (14)	9 (8)	
Infective	2 (4)	4 (4)	
Obstruction	4 (7)	3 (3)	
Hypertensive	1 (2)	5 (5)	
Interstitial	0 (0)	6 (6)	
Vascular	1 (2)	1 (1)	
Unknown	7 (13)	5 (5)	
Systolic BP (mmHg)	128 ± 17	126 ± 14	0.431
Diastolic BP (mmHg)	78 ± 11	77 ± 10	0.710
Haemoglobin (g/L)	138 ± 14	132 ± 22	0.057
Total cholesterol (mg/dL)	90 ± 18	90 ± 18	0.575
NTproBNP (pg/mL)	55 [34–93]	59 [17–161]	0.656
Immunosuppression usage *n* (%)	16 (29)	22 (21)	0.326
Anti-hypertensive usage *n* (%)	45 (82)	83 (80)	0.822
Statin usage *n* (%)	24 (46)	39 (37)	0.388
ECG *n* (%)			
Q waves	3 (5)	4 (4)	0.931
T inversion	5 (9)	12 (11)	0.789

Continuous variables are presented as mean ± SD if normally distributed or medians and [25th–75th percentile] for skewed variables. Categorical variables are presented as *n* (valid %). Significant *p* values are bold

*BMI* Body mass index, *BP* blood pressure, *eGFR* estimated glomerular filtration rate. *GN* Glomerulonephritis. *KDIGO* Kidney Disease Improving Global Outcomes. *LGEpos* Patients with late gadolinium enhancement. *LGEneg* Patients without late gadolinium enhancement

### CMR data

Data are presented in Table 2. There were no differences in RV or LV volumes, function or mass between LGEpos and LGEneg subjects.

**Table 2:** Cardiac MRI data according to presence or absence of late gadolinium enhancement

	**LGEpos*n*= 55**	**LGEneg*n*= 104**	***P*value**
LVEDVI (ml/m^2^)	61 ± 12	59 ± 12	0.484
LVESVI (ml/m^2^)	19 ± 8	18 ± 7	0.336
LVSV (ml)	82 ± 16	77 ± 17	0.095
LVEF (%)	68 ± 10	70 ± 8	0.382
LVMI (g/m^2^)	66 ± 14	62 ± 14	0.193
RVEDVI (ml/m^2^)	67 ± 14	68 ± 13	0.690
RVESVI (ml/m^2^)	28 ± 11	31 ± 10	0.057
RVSV (ml)	78 ± 17	74 ± 16	0.219
RVEF (%)	62 ± 9	61 ± 7	0.405
LVH	4 (7.3)	4 (3.8)	0.449

Continuous variables are presented as mean ± SD if normally distributed or medians [25-75th percentile] for skewed variables. Categorical variables are presented as n (valid %). Significant *p* values are bold

*LVEDVI* Left ventricular end-diastolic volume index. *LVESVI* Left ventricular end systolic volume index. *LVSV* Left ventricular stroke volume. *LVEF* Left ventricular ejection fraction. *LVMI* Left ventricular mass index. *LVH* Left ventricular hypertrophy. *RVEDVI* Right ventricular end diastolic volume index. *RVESVI* Right ventricular end systolic volume index. *RVSV* Right ventricular systolic volume. *RVEF* Right ventricular ejection fraction

### Patterns and quantification of LGE

**Fig. 2:**
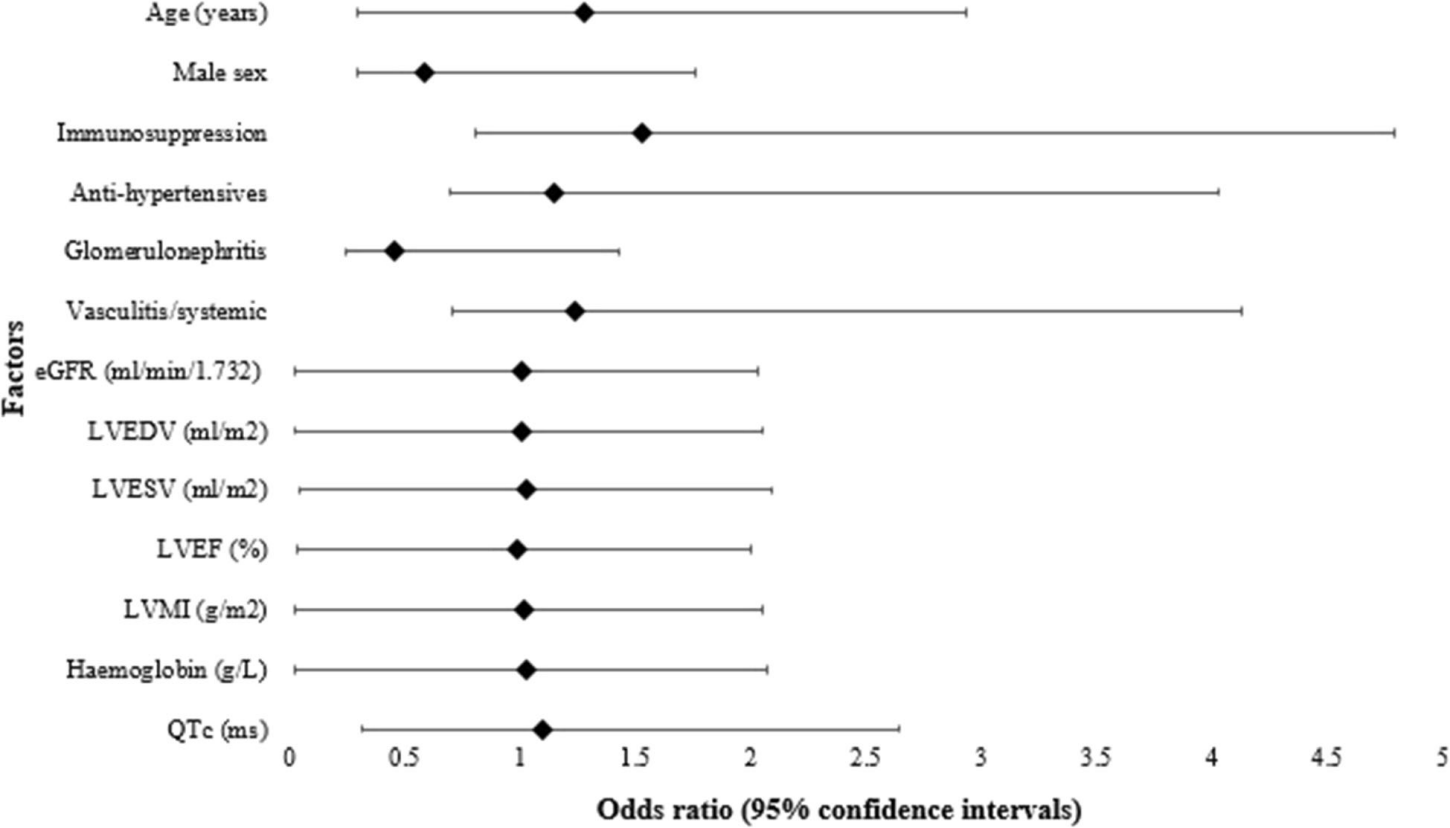
Predictors of LGE in CKD. Forest plot demonstrating the odds ratio (diamond marker) and 95% confidence intervals of all the factors which may influence the presence of late gadolinium enhancement on univariate analysis. The odds ratio (OR) is defined per 10 unit change. I.e. for age, OR given for per 10 years. Binary logistic regression was used for analysis with the dependent variable as the presence of LGE. All *p* values were more than 0.05 indicating there was no significant factor which predicts the presence of LGE on CMR. eGFR: Estimated glomerular filtration rate. LVEDVI: Left ventricular end diastolic volume, indexed. LVESVI: Left ventricular systolic volume, indexed. LVEF: Left ventricular ejection fraction. LVMI: Left ventricular mass index. LVH: left ventricular hypertrophy. QTc: Corrected QT interval

Patterns of LGE were RVIP *n* = 28/55 (50.9%), mid wall *n* = 18/55 (32.7%), sub-endocardial *n* = 5/55 (9.1%) and sub-epicardial *n* = 4 (7.3%). Examples of these patterns are presented in Fig. 1. There were no differences in demographic data, eGFR, blood pressure, ventricular function or mass between LGEpos and LGEneg subjects (Tables 1 and 2) or between the different patterns of LGE in positive patients (see Additional file 1: Table S2). Sub-group analysis of LGEpos with exclusion of RVIP patterns (*n* = 27), demonstrated higher LV mass 69 g/m^2^ vs 63 g/m^2^*p* = 0.029 and lower LVEF compared with LGEneg (69% vs. 66% *p* = 0.05). No specific pattern of LGE was associated with a defined aetiology. On multivariate modelling including demographics, CMR parameters and electrocardiogram (ECG) changes as variables, it was not possible to identify independent predictors of LGE (see Fig. 2).

The diffuse and RVIP patterns of LGE limited quantification (38/55 patients) but accounted for a very small percentage of overall mass, 1.25 [0.67–2.02] %. Intra-observer variability (of 10 patients) gave an absolute mean bias of 0.46% ± 0.43%, interclass correlation coefficient 0.98 (95% confidence interval 0.91–0.99). Inter-observer variability (of 10 patients) gave an absolute mean bias of 0.46 ± 0.80%, interclass correlation coefficient 0.97 (95% confidence interval 0.90–0.99).

Eight subjects with LGEpos had abnormal ECGs (in the absence of known CVD) with varied renal aetiology. Three patients had pathological Q waves; two had RVIP LGE with inferior Q-waves and one subject had sub-endocardial LGE pattern corresponding to anterior Q-waves. Five patients had T wave inversion; three had RVIP LGE, one had mid wall and one had a sub-endocardial pattern.

### Outcome measures

**Fig. 3:**
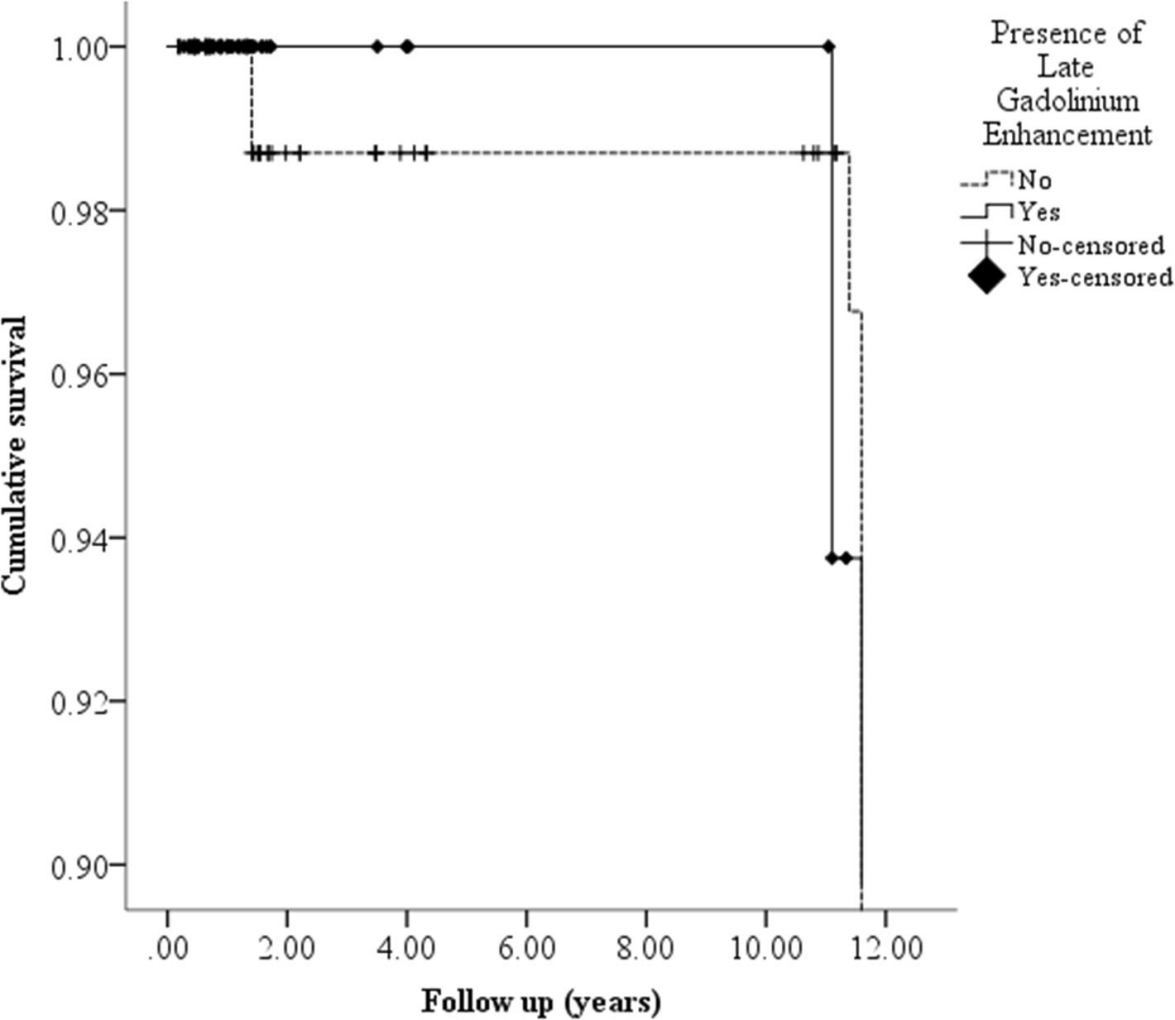
Kaplan- Meier curve of survival comparisons. A Kaplan-Meier curve demonstrates that there were very few events within those with and without LGE. There was no significant difference in survival between the two groups (Log Rank Mantel Cox and Breslow tests were non-significant at *p* = 0.154 and *p* = 0.209 respectively)

There were 15/159 patient deaths over follow up (9%); *n* = 6 (10.9%) in LGEpos patients and *n* = 9 (8.5%) in LGEneg. The cause of death was ascertained in 10 patients; 6 deaths were due to advanced carcinoma, one patient died of pneumonia, one patient died of acute cardiac failure secondary to ischaemic and hypertensive cardiac disease, one patient died of myocardial infarction and one patient died of a brain stem haemorrhage secondary to hypertension. A Kaplan Meier curve for all-cause mortality did not demonstrate a significant difference in mortality between the two groups (see Fig. 3). Cardiovascular morbidity was also low in this cohort (7%) with no difference between LGEpos and LGEneg; cardiac events (including heart failure) *n* = 5 vs. *n* = 2, stroke *n* = 1 vs. *n* = 2 and peripheral vascular disease *n* = 1 vs. *n* = 1. Hospital admissions occurred in 51 subjects (32%) although 26 of these were renal related admissions and only 4 subjects were admitted with cardiac causes (all those with chest pain were admitted for investigations and subsequent angiograms). The remaining admissions were non-cardiac and included admissions for a range of medical and surgical complaints including seizures, urinary tract infections, hernias and fractures.

### Subgroup analysis

Further analysis was conducted to compare subgroups of LGE in an attempt to determine whether those with RVIP patterns had a different risk profile in CKD to other patterns of LGE. When comparing the patients with LGEneg or RVIP LGE (*n* = 132) to all other remaining patterns of LGE (*n* = 27) there was no significant difference in survival (Log rank *p* = 0.316, Breslow *p* = 0.397). There was also no difference in cardiac events, heart failure, stroke or peripheral vascular disease. Similarly when those with RVIP (*n* = 28) were compared to all other types of LGE (*n* = 27) there was no difference in survival (Log rank *p* = 0.404, Breslow *p* = 0.299) cardiac events, heart failure, stroke or peripheral vascular disease.

## Discussion

This large retrospective CMR study has examined the prevalence, patterns and clinical significance of LGE in patients with pre-dialysis CKD. Over a third of subjects had evidence of LGE with normal LVEF and low rates of LVH. However, LGE, did not predict adverse cardiac events and did not offer incremental risk stratification in this population. This finding may reflect that over half of the LGE was focal at the right ventricular insertion points, a pattern shown to be associated with a better prognosis and correlated on histology with myocardial disarray, increased collagen and fat between fibre bundles but not replacement fibrosis [17]. Only 18/159 (11%) patients had a mid-wall / diffuse pattern in keeping with myocardial interstitial fibrosis from a non-coronary aetiology which is far lower than the reported ~ 30% seen in non-ischaemic dilated cardiomyopathy (NI-DCM) where LGE has prognostic stratification [18]. CMR plays an increasingly important role in characterizing heart disease in CKD and an appreciation of the myocardial changes frequently present are important for clinicians. The observed low cardiac event rate over intermediate follow up might also offer a degree of reassurance to clinicians when assessing CKD patients without high risk CV markers such as diabetes or proteinuria.

RVIP-LGE has been reported not only in NI-DCM, pulmonary hypertension and hypertrophic cardiomyopathy but also in athletes [19–21]. It is thought to represent plexiform fibrosis related to interdigitation of RV and LV fibers as these form the septum rather than myocardial fibrosis and represents only 1–3% of overall LV mass [22]. The natural history of RVIP-LGE in CKD remains unknown. Our finding of isolated RVIP-LGE and a more benign phenotype is supported in a recent prospective observational study examining patterns of LGE in NI-DCM with severe LV impairment and a mean LVEF of 25% over 46 months follow up. RVIP-LGE was present in 14% of the cohort and was associated with higher systolic and diastolic blood pressure but did not increase the risk of adverse cardiac event or arrhythmic events compared to patients with no LGE. Furthermore, there was a lower incidence of heart failure than observed with myocardial LGE [17]. In pulmonary hypertension, observational studies have also shown that RVIP-LGE does not predict mortality in contrast to more extensive LGE extending into the interventricular septum [21].

In contrast, there are extensive data to support the role of myocardial LGE as a marker of risk in ischaemic and non-ischaemic cardiomyopathies even when the LVEF is normal [1, 18]. Myocardial LGE correlates on histology with expansion of the extracellular space, increased collage deposition and ultimately irreversible fibrosis [23]. The latter serves as a substrate for ventricular arrhythmias and promotes adverse ventricular remodelling [23]. In CKD, histological data showing myocardial interstitial fibrosis and disarray on endomyocardial biopsy was first reported in the 1990s and the subsequent use of LGE-CMR demonstrated 14% of patients with ESKD had evidence of mid-wall reparative fibrosis [7, 8]. Unlike other disease cohorts, large outcome studies demonstrating the prognostic role of LGE in CKD have not followed and further gadolinium LGE-CMR studies have been restricted since 2006 with regulatory changes to reflect the reported association between gadolinium-based contrast agents used for magnetic resonance imaging (MRI) procedures and nephrogenic systemic fibrosis (NSF).

The largest study to date in CKD was a retrospective analysis of 966 “all comers” undergoing LGE-CMR between 2006 and 2008 with a serum creatinine measured within 30 days of CMR. Patients were stratified according to eGFR greater or less than 70 ml/min/1.73m^2^ [24]. The authors demonstrated lower eGFR to be associated with more LGE even after adjustment for demographics, coronary disease and diabetes. Stratification of survival based on eGFR demonstrated higher mortality with LGEpos and eGFR< 70 ml/min1.73m^2^ with a hazard ratio of 1.8 (1.07–3.04, *p* = 0.03) compared to LGEneg subjects [24]. This cohort was a very different population to our study. Firstly, subjects were ‘all comers’ who had all undergone CMR for clinical reasons and most subjects had coronary artery disease (54%) or diabetes (16%) [24]. This contrasts with those recruited to our study, the majority of whom were identified a priori based on the presence of renal disease confirmed on biopsy/imaging and without cardiovascular disease. Secondly, the predominant pattern of LGE found in 88% was sub-endocardial ‘coronary’ infarct, suggesting that the study by Dandamudi et al. was of a cohort of clinical patients with ischaemic heart disease and mild secondary renal impairment (mean eGFR 73 ml/min/1.73m^2^) [24]. The ratio of sub-endocardial LGE in their study is disproportionate not only to the amount of a non-coronary pattern seen in our study but also that in the landmark study by Mark et al. [7].

There are several potential reasons why LGE did not predict adverse outcomes in our study of CKD contrary to the near universal finding in non-ischaemic cardiomyopathies where “LGE is bad”. Firstly, our cohort was selected as part of research trials to investigate the impact of CKD on changes in myocardial structure and function, and hence specifically excluded subjects with diagnosed cardiovascular disease and diabetes. Secondly, although the majority of our cohort were male, hypertensive and many were prescribed lipid lowering therapy, most also had only early stage CKD and the overall CV adverse event rate was low. It is possible that the follow-up period was too short to identify adverse outcomes related to myocardial LGE but it may also be that the impact of myocardial fibrosis in CKD may be most adverse in those with an incremental number of risks, perhaps including sub-clinical ischaemia. The latter hypothesis is consistent with the recent scientific statement from the American Heart Association, concluding that multiple mechanisms contribute to the progression of uremic cardiomyopathy rather than this being driven solely by a decline in eGFR [25]. The impact of multiplicative CV risk factors including hypertension, calcium-phosphate metabolism and diabetes on LGE prevalence remains to be established in higher risk CKD cohorts.

### Limitations and clinical implications

There are several limitations of our study; the data were retrospectively acquired and are observational from pre-defined and selected research cohorts. The small sample size, long follow up and small number of events makes this study vulnerable to type two statistical error. The low number of adverse clinical outcomes is partly due to a highly selected patient group and single centre data which might have precluded identification of an independent association of adverse events with LGE. It is also possible that clinical events have been missed in patients presenting to other hospitals. We acknowledge a selection bias in our cohort. Patients recruited into our studies are a highly selected group with controlled blood pressure and without diabetes and many comorbidities. The aetiology is also most commonly primary glomerulonephritis or adult polycystic kidney disease. We also acknowledge that the duration of CKD would be expected to affect the prevalence of myocardial disease. However, it was not possible to reliably established this vintage in pre-dialysis patients many of whom were only referred from primary care at a stage of more rapid decline or falling below eGFR referral cut offs. Further studies will require the enrolment of unselected subjects with CKD and longer follow up times to assess the prognostic implications of LGE in CKD. It is noteworthy that we have no reported cases of NSF in over 10 years of CKD based research. However, precautions are taken to minimise dose (0.15–0.2 mmol/kg), newer low risk gadolinium agents are used (Gadovist®) and contrast is avoided in patients with acute kidney injury and when eGFR < 30 ml/min/1.73m^2^. The absence of any cases of NSF is reassuring but, our sample size is too small and NSF is too rare to draw conclusions.

Recruitment and data collection commenced before widespread availability of newer non-contrast T1 mapping CMR sequences. These sequences have undoubtedly allowed better detection of diffuse fibrosis which can be “missed” with LGE inversion recovery sequences due to limited spatial resolution [26]. Observational data in CKD cohorts have been consistent in demonstrating elevated native T1 times in early stage CKD and ESKD on dialysis [9, 27, 28]. However, to date there is not histological correlation with T1 in CKD. Indeed a recent proof of concept study correlating the extent of myocardial fibrosis on LGE with absolute native T1 signal failed to identify “cut off” thresholds in a validation model [29]. To date, the most comprehensive assessment of myocardial fibrosis and its clinical impact appears to be through combining multi-parametric CMR biomarkers of LGE, T1 and ECV. This approach was recently used in a cohort with aortic stenosis and successfully identified patients with worse LV remodelling, adverse blood biomarkers such as NT pro-BNP and worse functional capacity [30]. Continued restrictions on use of gadolinium in CKD means T1 mapping endpoints will potentially have increasing importance for longitudinal follow up and outcome prediction.

## Conclusions

LGE is common in patients with pre-dialysis CKD who do not have clinical ischaemic heart disease. The predominant pattern was in the RVIP distribution with lower rates of mid-wall fibrosis than is reported in non-ischaemic cardiomyopathy. In this low risk, stable cohort, rates of CV events were low and LGE did not predict an adverse prognosis. This study does not exclude an association between mid-wall/myocardial LGE and adverse prognosis in those with advanced CKD and multiple risk factors.

## Supplementary information

**Additional file 1: Table S1.** A summary of details of each study included in this cohort. **Table S2.** Demographics and CMR data according to patterns of LGE.

## Data Availability

The data set used during this study are available from the corresponding author on reasonable request.

## Abbreviations

BMI: Body mass index
CKD: Chronic kidney disease
CMR: Cardiovascular magnetic resonance
CV: Cardiovascular
ECG: Electrocardiogram
eGFR: Estimated glomerular filtration rate
ESKD: End stage kidney disease
KDIGO: Kidney disease improving global outcomes
LGE: Late gadolinium enhancement
LGE-CMR: Late gadolinium cardiovascular magnetic resonance
LGEneg: Patients without late gadolinium enhancement
LGEpos: Patients with late gadolinium enhancement
LV: Left ventricular
LVEDVI: Left ventricular end diastolic volume index
LVEF: Left ventricular ejection fraction
LVESVI: Left ventricular end systolic volume index
LVH: Left ventricular hypertrophy
LVMI: Left ventricular mass index
LVSV: Left ventricular stroke volume
MDRD: Modification of diet in renal disease
MRI: Magnetic resonance imaging
NI-DCM: Non ischaemic dilated cardiomyopathy
NSF: Nephrogenic systemic fibrosis
NTproBNP: N-terminal pro b-type natriuretic peptide
RV: Right ventricular
RVEDVI: Right ventricular end diastolic volume index
RVEF: Right ventricular ejection fraction
RVESVI: Right ventricular end systolic volume index
RVIP: Right ventricular insertion point
RVSV: Right ventricular systolic volume
