# Supplementary material for: Myocardial characterization in pre-dialysis chronic kidney disease: a study of prevalence, patterns and outcomes
Source: BMC Cardiovasc Disord. 2019 Dec 16;19:295. doi: 10.1186/s12872-019-1256-3 (PMC6916031; doi:10.1186/s12872-019-1256-3)
Supplement: Supplementary file 1 — Additional file 1: Table S1. A summary of details of each study included in this cohort. Table S2. Demographics and CMR data according to patterns of LGE. [file 12872_2019_1256_MOESM1_ESM.docx]

**Supplementary data**

**Contents**

Table S1: A summary of details of each study included in this cohort.

Table S2: Demographics and CMR data according to patterns of LGE.

**Table S1:** A summary of details of each study included in this cohort.

| **Study name and ethical approval** | **Exclusion/Inclusion criteria** | **Methods of excluding ischaemic heart disease** | **Gadolinium dose, generic brand** | **Scan parameters** | **Patient demographics of those recruited** |
| --- | --- | --- | --- | --- | --- |
| Chronic Renal Impairment in Birmingham II  CRIB II [1]  Approved by South Birmingham Local Research Ethics Committee | **Inclusion:**   - CKD stage 2 (eGFR 60 to 89 ml/min/1.73 m^2^ with other evidence of kidney disease. - Stage 3 (eGFR 30 to 59 ml/min/1.73 m^2^), and stage 4 (15 to 29 ml/min/1.73 m^2^)   **Exclusion:**   - Cardiovascular disease or symptoms - Valvular heart disease - AF - Diabetes | Clinical history, ECG, Echocardiography | Magnevist® 0.2mmol/kg | 1.5 Tesla  LV function  LV mass  Aortic distensibility  LGE | Average age: 53yrs  ~60% male  Average eGFR: 50ml/min/1.73m^2^  Average office blood pressure: 130/77 mmHg  Left ventricular hypertrophy 8%  69% taking angiotensin converting enzyme inhibitors |
| Diffuse fibrosis in CKD.[2]  Approved by the National Research Ethics Service—South Birmingham (12/WM/0250) | - As in CRIB II   Healthy controls   - Normotensive or well controlled hypertension - No history of CVD - Renal function within expected range for age. | Clinical history, ECG | Gadovist ® 0.15mmol/Kg | 1.5 Tesla  LV function  LV mass  T1 mapping  LGE | Average age: 57yrs  ~56% male  Average eGFR: 50ml/min/1.73m^2^ (CKD cohort)  Average office blood pressure: 127/69 mmHg  Left ventricular hypertrophy 5%  >80% taking angiotensin converting enzyme inhibitors or angiotensin receptor blockers  37% Glomerular nephropathy. 21% Adult polycystic kidney disease. |
| Left Ventricular Disease in Chronic Kidney Disease.  CKD-Fibrosis  NCT03176862  Approved by the National Research Ethics Service – East Midlands (15/EM/0280) | **Inclusion:**   - >18 years of age. - CKD stage 2 to 5 plus other evidence of kidney disease i.e. proteinuria.   **Exclusion**   - Diabetes - Any previous cardiovascular disease - Cerebrovascular disease. - Peripheral vascular disease. - Pregnancy - Reno vascular disease. | Stress Echo Cardiopulmonary exercise test. | Gadovist ® 0.15mmol/Kg | 1.5 Tesla  LV function  LV mass  T1 mapping  LGE if eGFR >30ml/min | Average age: 52 yrs  ~60% male  Average office systolic blood pressure 134mmHg  93% on anti-hypertensives  Majority of patients had primary glomerulonephritis or adult polycystic kidney disease |

AF; Atrial fibrillation. CKD; Chronic Kidney Disease. eGFR; estimated glomerular filtration rate. ECG; Electrocardiogram

**Table S2:** Demographics and CMR data according to patterns of LGE.

|  | **RVIP**  **n=28** | **Mid-wall**  **n=18** | **Subendocardial**  **n=5** | **Subepicardial**  **n=4** |
| --- | --- | --- | --- | --- |
| Age (years) | 57 ± 13 | 57 ± 10 | 55 ± 14 | 59 ± 9 |
| Male sex *n* (%) | 21 (75) | 11 (61) | 4 (80) | 2 (50) |
| BMI (kg/m^2^) | 28 ± 3 | 27 ± 3 | 30 ± 2 | 30 ± 7 |
| eGFR (ml/min/1.73m^2^) | 55 ± 17 | 56 ± 13 | 41 ± 6 | 58 ± 9   \|  \| Total n=161 \| LGEpos  n=55 \| LGEneg  n=106 \| P value \| \| --- \| --- \| --- \| --- \| --- \| \| LVEDV (ml/m^2^) \| 60 ± 13 \| 61 ± 12 \| 60 ± 14 \| 0.78 \| \| LVESV (ml/m^2^) \| 19 ± 9 \| 19 ± 8 \| 19 ± 9 \| 0.75 \| \| LVSV (ml) \| 79 ± 16 \| 82 ± 16 \| 78 ± 17 \| 0.09 \| \| LVEF (%) \| 69 ± 9 \| 68 ± 10 \| 69 ± 8 \| 0.56 \| \| LVMI (g/m^2^) \| 64 ± 14 \| 66 ± 14 \| 63 ± 14 \| 0.28 \| \| RVEDV (ml/m^2^) \| 68 ± 14 \| 67 ± 14 \| 68 ± 14 \| 0.62 \| \| RVESV (ml/m^2^) \| 30 ± 11 \| 28 ± 11 \| 31 ± 10 \| 0.05 \| \| RVSV (ml) \| 75 ± 16 \| 78 ± 17 \| 74 ± 16 \| 0.22 \| \| RVEF (%) \| 61 ± 7 \| 62 ± 9 \| 61 ± 7 \| 0.39 \| \| LVH \| 9 (5.6) \| 4 (7.3) \| 5 (4.7) \| 0.49 \| |
| Systolic BP (mmHg) | 125 ± 16 | 130 ± 16 | 133 ± 26 | 128 ± 8 |
| Diastolic BP (mmHg**)** | 76 ± 11 | 82 ± 10 | 76 ± 13 | 76 ± 11 |
| Haemoglobin (g/L) | 135 ± 14 | 140 ± 13 | 150 ± 20 | 138 ± 16 |
| Total cholesterol (mmol/L) | 5.1 ± 1 | 5 ± 1 | 4 ± 1.5 | 5.1 ± 0.7 |
| Immunosuppression usage *n (%)* | 8 (30) | 6 (35) | 1 (20) | 1 (25) |
| Anti-hypertensives usage *n (%)* | 21 (78) | 15 (88) | 5 (100) | 4 (100) |
| Statin usage  *n (%)* | 15 (53) | 6 (40) | 3 (60) | 1 (25) |
| ECG *n (%)* |  |  |  |  |
| Q wave | 2 (7) | 0 (0) | 1 (20) | 0 (0) |
| T wave inversion | 3 (11) | 1 (6) | 1 (20) | 0 (0) |
| CMR data |  |  |  |  |
| LVEDV (ml/m^2^) | 61 ±11 | 59 ± 11 | 74 ± 14 | 54 ± 13 |
| LVESV (ml/m^2^) | 18 ± 6 | 18 ± 5 | 37 ± 12 | 16 ± 5 |
| LVSV (ml) | 85 ± 15 | 78 ± 17 | 78 ± 16 | 80 ± 11 |
| LVEF (%) | 71 ± 7 | 68 ± 10 | 51 ± 8 | 72 ± 7 |
| LVMI (g/m^2^) | 62 ± 13 | 67 ± 14 | 79 ± 16 | 67 ± 8 |
| RVEDV (ml/m^2^) | 68 ± 14 | 67 ± 16 | 63 ± 7 | 64 ± 4 |
| RVESV (ml/m^2^) | 27 ± 10 | 29 ± 15 | 29 ± 5 | 24 ± 3. |
| RVSV (ml) | 80 ± 17 | 74 ± 16 | 73 ± 23 | 77 ± 10 |
| RVEF (%) | 62 ± 7 | 61 ± 11 | 60 ± 9 | 59 ± 10 |
| LVH *n (%)* | 1 (3.6) | 2 (11) | 1 (20) | 0 (0) |

Continuous variables are presented as mean ± SD if normally distributed or medians and [25^th^-75^th^ percentile] for skewed variables. Categorical variables are presented as *n* (valid %). Significant p values are bold. BMI: Body mass index. BP: blood pressure, CMR; Cardiovascular magnetic resonance. eGFR: estimated glomerular filtration rate, KDIGO: Kidney Disease Improving Global Outcomes. LVEDVI: Left ventricular end-diastolic ventricular volume index. LVESVI: Left ventricular end systolic ventricular volume index. LVSV: Left ventricular stroke volume. LVEF: Left ventricular ejection fraction. LVMI: Left ventricular mass index. LVH: Left ventricular hypertrophy. RVEDVI: Right ventricular end diastolic volume index. RVESVI: Right ventricular end systolic volume index. RVSV: Right ventricular systolic volume. RVEF: Right ventricular ejection fraction.

*There was no significant differences between groups.*

**References**

1. Edwards NC, Steeds RP, Stewart PM, Ferro CJ, Townend JN: **Effect of Spironolactone on Left Ventricular Mass and Aortic Stiffness in Early-Stage Chronic Kidney Disease: A Randomized Controlled Trial**. *Journal of the American College of Cardiology* 2009, **54**(6):505-512.

2. Edwards NC, Moody WE, Yuan M, Hayer MK, Ferro CJ, Townend JN, Steeds RP: **Diffuse Interstitial Fibrosis and Myocardial Dysfunction in Early Chronic Kidney Disease**. *American Journal of Cardiology 2015*, **115**(9):1311-1317.
